# Appropriateness of admission to two intensive care units at alexandria main university hospital, egypt

**DOI:** 10.1186/2197-425X-3-S1-A478

**Published:** 2015-10-01

**Authors:** R Abuyadek, H Helmy, AM Fayed, M Abdelwahab, D Fouad

**Affiliations:** Alexandria University / High Institute of Public Health, Health Administration and Behavioral Sciences, Alexandria, Egypt; Alexandria University, Faculty of Medicine, Critical Care Medicine, Alexandria, Egypt; Alexandria University / High Institute of Public Health, Department of Biostatistics, Alexandria, Egypt

## Introduction

Resource allocation and problem of inappropriate admissions in ICUs has been a global concern. No studies were found in Egypt to investigate it.

## Objectives

Assessment of appropriateness of admission to two intensive care units, through assessment of; adherence to guidelines of ICU admission recommended by Society of Critical Care Medicine (SCCM) [[1]], severity of illness of admitted patients using APACHE II [[2]] score, utilization of ICU resources and outcome of ICU admission [[3]].

## Methods

The study was conducted in ICUs of Alexandria Main University Hospital, medical records of the adult patients admitted to the ICUs from 2013-2014, were the target population; 324 patients were included. Data collection methods; concurrent review in ICUs during patient admission, to capture day one clinical data for calculation of APACHE II score and reviewing day one ICU specific interventions received and retrospectively in medical record department after patient discharge from the hospital for retrieval of data related to outcome of ICU admission episode.

## Results

The demographic characteristics and administrative data of the patients admitted were studied. Regarding age the highest percent of cases (43.8%) were belonging to age category 50- < 70 years old. Regarding sex, 59.6% were males. Most common source of admission was ER (89.5%), About 92.9% of the sample was medical admissions. Approximately 42% of them were admitted in the night shift.

Nearly, all of them were adherent to diagnosis model of SCCM guidelines of ICU admission and approximately 75% were adherent to objective parameters model of SCCM guidelines. Mean APACHE II score of admitted cases was 13.84 ± 6.861. Outcome of ICU admission episode; 39.8% of sample patients were died, while 33.3 % were transferred to hospital ward, about 22% were discharged against medical advice. Use of ICU specific interventions in the first 24 hours of admission was assessed. About 18% of cases didn’t receive ICU specific treatment in the first 24 hours of admission. A comparison was done between patients who didn’t receive ICU active treatment in the first 24 hours of admission to ICU and those who received regarding age, APACHE II score, ICU length of stay and outcome of ICU admission.

## Conclusions

Selection of patients is nearly appropriate, but rationing towards selected groups is recommended.

## Grant Acknowledgment

No grants received
